# Poor insight and future thinking in early dementia limit patient projections of potential utility of technological innovations and advanced care planning

**DOI:** 10.3389/fmed.2023.1123331

**Published:** 2023-03-13

**Authors:** Jaye E. McLaren, Marlena H. Shin, Lauren R. Moo

**Affiliations:** ^1^New England Geriatric Research Education and Clinical Center, Veterans Affairs Bedford Healthcare System, Bedford, MA, United States; ^2^Center for Healthcare Organization and Implementation Research, Veterans Affairs Boston Healthcare System, Boston, MA, United States; ^3^Center for Healthcare Organization and Implementation Research, Veterans Affairs Bedford Healthcare System, Bedford, MA, United States; ^4^Harvard Medical School, Boston, MA, United States; ^5^Department of Neurology, Massachusetts General Hospital, Boston, MA, United States

**Keywords:** cognition, technology, geriatrics, qualitative, insight, patient-centered

## Abstract

**Introduction:**

Cognitive psychology posits that thinking about the future relies on memory such that those with memory impairment may have trouble imaging their future technology and other needs.

**Methods:**

We conducted a content analysis of qualitative data from interviews with six patients with MCI or early dementia regarding potential adaptations to a mobile telepresence robot. Using a matrix analysis approach, we explored perceptions of (1) what technology could help with day-to-day functioning in the present and future and (2) what technology may help people with memory problems or dementia stay home alone safely.

**Results:**

Very few participants could identify any technology to assist themselves or other people with memory problems and could not provide suggestions on what technology may help them stay home alone safely. Most perceived that they would never need robotic assistance.

**Discussion:**

These findings suggest individuals with MCI or early dementia have limited perspectives on their own functional abilities now and in the future. Consideration of the individuals’ diminished understanding of their own future illness trajectory is crucial when engaging in research or considering novel technological management solutions and may have implications for other aspects of advanced care planning.

## 1. Introduction

Alzheimer’s disease and related dementias (ADRD) are neurodegenerative diseases that permanently alter neural processes impacting not only memory and other aspects of cognition but functional activities of daily living. Over 57 million people live with ADRD today and as many as 152 million may do so by 2050 (1). Brain imaging and other biomarker research demonstrate neurodegeneration is present long before individuals experience challenges in their everyday life (2). Memory loss, one of the hallmarks of ADRD, becomes more apparent as the disease progresses. Individuals initially have trouble with new learning and remembering recent events, but as their dementia progresses, they become less able to call upon more remote past experiences (3).

Research into the cognitive and neural underpinnings of memory has shown that this process is an integral part of picturing future experiences. Memory is broadly categorized into semantic memory (facts, concepts) or episodic memory (experiences or life events). Episodic future thinking, as defined by Schacter et al. (4) refers to “the capacity to imagine or simulate experiences that might occur in one’s personal future” (4 p. 41) and there is “a distinct role for episodic retrieval in imagining future experiences.” (4 p. 44) Future thinking is understood to reflect the retrieval and recombination of past experiences, both internal details (what/when/where) and external details (semantic details and commentary), reimagined into a new, novel experience (4). That said, what if one’s ability to accurately remember the past has been disrupted? Previous research has found that patients with Alzheimer’s disease provided less details overall in their description of projected future events, suggesting a deficit in future thinking (5, 6). Further, when assessing future thinking as it relates to functional ability and completing instrumental activities of daily living independently (IADLs) (e.g., planning for travel, or managing medications or finances), researchers identified a significant connection between the ability to provide sufficient details in a future thinking task and independent completion of IADLs. It is suggested that future thinking may be a crucial element in the cognitive process that helps individuals conceptualize and sequence the steps necessary to successfully perform daily activities (7). Therefore, the challenges that mild cognitive impairment (MCI) and early ADRD pose on future thinking demonstrate the potential for negative clinical implications as the disease progresses.

Although those with MCI or early ADRD are still able to communicate and complete many functional tasks independently, a key question is, do they have the ability to accurately project into the future and imagine the supports they may need to maximize functional ability, safety, and independence as their dementia progresses? Policy planners and technology designers often assume that older adults are either not comfortable with technology or that technology will provide the majority of solutions for our growing number of older adults; however, the lived experiences of this population are more nuanced. Continued advances in and changes to technology coupled with memory decline may further limit the person with MCI or early ADRD’s ability to imagine how technology could aid with functional independence. Per patient-centered care and user-centered design principles, it is important to involve target populations as study participants (8). Those with MCI or early ADRD are still able to communicate, and thus, should be asked about their thoughts and offer feedback on technology design. This poses challenges though when the population in question has cognitive decline and may demonstrate challenges in future thinking and imagining their future needs, especially related to technological assistance.

In this brief report, our goal is to offer insights into the key question posed above. To do this, we explore participants’ perceptions of what technology could assist those with MCI or early ADRD in the future as their dementia progressed, as well as what technology may be needed in the future to help them stay home alone safely. Exploring these results on technological needs may help to provide additional perspectives into future thinking in dementia and inform clinical as well as technology research approaches.

## 2. Methods

For this brief report, we analyzed qualitative interview data collected in a previous study (July to August 2019) in which we obtained feedback on possible adaptations to a mobile telepresence robot and the robot’s potential utility from stakeholder groups, including patients with MCI or early ADRD (9). Approval from the Institutional Review Board at the Veterans Affairs (VA) Bedford Healthcare System (approval number 110818) was received.

### 2.1. Recruitment and data collection

Recruitment was completed at the VA Bedford and inclusion criteria for the specific population of interest in this brief report included patients: (1) who were ages 50 and older with a diagnosis of MCI or early ADRD in their medical chart and living in the community (not a nursing home or assisted living facility); (2) who were routinely left home alone for periods of at least 4 h; and (3) who had no indication of incompetence in their medical record (9). Potential participants were identified as having either MCI or ADRD based on the terminology used in the most recent physician note of neurological function in their medical record from either the dementia clinic or outpatient primary care clinics at the Bedford VA. Researchers did not complete additional independent assessments of cognition for this study.

For those patients who chose to opt-in to the study, we obtained informed consent for participation as well as to audio record prior to beginning the interviews. Six patients participated in a 60–90 min semi-structured qualitative interview during which participants were shown a video of a remotely navigable mobile telepresence robot and we elicited opinions regarding their technology use and feedback about the robot. The specific robot depicted in the video participants viewed was a wheeled, upright robot approximately 120 cm tall with a top-mounted screen and was seen to be navigated through an elementary school under the remote guidance of a student who was homebound. We explained that mobile telepresence robots can be used in a wide variety of settings to facilitate communication between individuals who are not co-located. This allows for increase social connection and exchange and has potential implications for healthcare delivery and support. In addition, as part of the interview, we asked participants about: (1) What kind of technology do you think could help you with day to day functioning? and (2) What are the main things you think might prevent people with memory problems or dementia from staying home alone safely? Participants were compensated $50 for participating in the interview and an additional $30 stipend if they traveled to attend the interview in person. Additional details of the recruitment process, the mobile telepresence robot, the data collection process, and participant demographics are described in our previous study (9).

### 2.2. Data analysis

For this brief report, we conducted a content analysis using the reports that had been generated from the coding of the qualitative interview data in our previous study, which further describes our process (9). In this analysis, we focus on two specific areas of interest: participants’ perceptions of technology to (1) help with day-to-day functioning and (2) help people with memory problems or dementia stay home alone safely. We used a matrix analysis approach to organize, review, and assess the data in the code reports for these areas of interest (10). Our goal, when analyzing the content of the data in the matrix, was to explore any perceptions offered by participants that could provide insight into technology use and future thinking in patients with memory problems or dementia.

## 3. Results

### 3.1. Perceptions of what technology could help with day-to-day functioning

When asked about what potential technology could help with day-to-day functioning, only 2 out of 6 participants identified technology that would assist with memory problems commonly experienced by this population.

“I do use my home Google for reminders all the time…I use that a lot…I’d be lost without it. I don’t see how I functioned years [ago] because back then everything was paper and pencil.” Participant D

“I know a friend of mine had somebody on his phone that he asked questions to and they answered them. I don’t have that on my phone.” Participant F

Most participants discussed wanting technology to help address physical limitations of a task (e.g., assistance with IADLs like housework and picking up medication) vs. technology to address cognitive limitations (e.g., technology similar to Siri or Google Home for reminders).

“Working around the house [and] getting things done.… Something to run around the house and clean.” Participant A

“I suppose if somebody is disabled and can’t walk and can’t do that if there are chores that [a robot] can do.” Participant C

All participants perceived that the technology they had at the moment was sufficient both now and for the future.

We observed that participants had challenges in articulating their future technology needs. With further prompting from interviewers who provided a list of potential ways in which technology could assist with cognitive limitations (e.g., emergency help access, medication and schedule reminders, communication assistance with family and doctors, and social stimulation), 5 out of 6 participants were able to agree that, in general, some technology, especially if integrated into a mobile telepresence robot, may help with cognitive barriers experienced by individuals with MCI or early ADRD.

However, when interviewers asked participants about whether the mobile telepresence robot, in particular, could be helpful to them in the future, 4 out of the 6 participants responded that they, themselves, would not need robotic assistance at any time. Reasons included the lack of need for technology (e.g., a mobile telepresence robot) due to the presence of other family members as well as the lack of desire to learn how to use technology, such as a computer or the robot.

“I don’t think so. Not me personally because I won’t be in a situation where it will be necessary to have [a mobile telepresence robot]… I’ll be living with my children.” Participant C

“I don’t think it would be helpful at all. Not for me. I wouldn’t know how to use it. My wife would be fine, but me, no. It’s just I’m computer illiterate and have no desire to even learn how to use a computer.” Participant E

### 3.2. Perceptions of what technology may help people with memory problems or dementia stay home alone safely

When asked about what might prevent people with memory problems or dementia from staying home alone safely, participants mostly discussed physical issues that can prevent people from staying home alone safely (e.g., falling and tripping).

“Tripping on carpets. I go upstairs – half the time when I go up the stairs I fall going up the stairs than the down. That’s because my equilibrium gets off quite a bit and I forget.” Participant A

“Maybe for somebody that falls down or something like that and then can’t get up. I would say either falling down or not being able to get hold of help would be the most important if something came up where you couldn’t do it on your own.” Participant D

Even with further prompting, participants had challenges in brainstorming safety issues and often noted that they would have their caregivers’ help. In addition, they could not provide suggestions on what technology may help them stay home alone safely. Three out of six participants reported knowing someone close to them who had progressed through the stages of dementia; these participants were more likely to identify at least one potential technological solution for cognitive challenges.

“[It would be helpful to have a mobile telepresence robot that reminded people with ADRD] to take your medicines that you need on time and when and how much. And time to eat – [a mobile telepresence robot could be helpful to] remember that. I think people with Alzheimer’s and dementia forget to do those things. My dad had it and I remember seeing him and he forgot a lot so [a mobile telepresence robot] could help with that kind of a thing.” Participant C

## 4. Discussion

While our previous study (9) showed that those with MCI or early ADRD were able to provide overall feedback regarding the proposed assistive technology, when asked to imagine the assistance they may require in the future, they lacked future thinking capability in predicting their own future cognitive and functional abilities. Participants had a limited ability to independently identify potential cognitive challenges and suggest technology that could be of assistance for their future cognitive and functional declines. These findings demonstrate both a decrease in insight and suggest potential deficits in future thinking cognitive abilities, specifically the ability to project in the future as the disease progresses. This is consistent with cognitive psychology research linking impaired memory to deficits in future thinking abilities (5, 6).

Even those participants who reported having witnessed someone progress through all the stages of dementia had challenges in identifying potential technological solutions to address cognitive limitations. Findings presented in this report provide valuable insight into the mindset of persons with MCI or early ADRD and their limited perspectives on declines in function now and in the future. Results are consistent with previous research that identifies the clinical implications of individuals’ (lack of) self-awareness or understanding of the likely progression of their own illness (also known as anosognosia). These include overall safety considerations as well as aspects of advanced care planning (e.g., creating a living will, end-of-life planning, etc.) (11). Our research supports these findings and also highlights the importance of understanding these deficits when considering novel technological dementia management solutions.

Similarly, most participants verbalized a discomfort with technology and more specifically, the idea of using a computer or a machine in the future to assist with tasks that could be impacted by functional or cognitive decline. As dementia is a neurodegenerative disease, people with dementia will eventually experience decline and require assistance to safely complete daily tasks. Technology and telehealth can help people with dementia age in place longer (12, 13). Previous research supports our findings that older adults are generally not as interested in adopting and utilizing technology in their daily life (14, 15). Coupled with our findings that reveal a decrease in future thinking for those with MCI and early ADRD, it demonstrates the need to identify a “sweet spot” in time to introduce older adults to technology as early on in the disease process as possible in an effort to maximize the potential for technology comfort and adoption as cognition declines.

For technology developers, understanding how to best engage individuals with MCI or early ADRD in a participatory co-design process, such that the technology is patient-centered, is critical. Several recommendations have been made on what to do when using a participatory co-design process with people living with MCI or early ADRD regarding the utility of technological products, such as asking them how they would like to participate in the research, using supportive rather than offensive language, ensuring their emotional and physical safety, and being respectful, compassionate and tolerant when working with them (16). Technology developers as well as researchers should keep these recommendations in mind as they design and conduct their work as this can promote meaningful experiences across all who are involved. Our experiences in interviewing people living with MCI or early ADRD also support these suggestions. During the interviews, our study researchers utilized advanced interviewing techniques, such as rephrasing and clarifying questions as well as inquiring deeper about answers given, to increase engagement from participants. Given the challenges in their future thinking, this also required researchers to, for example, use supportive language use supportive language and remain respectful, compassionate, and tolerant of how participants were answering the questions. During each interview, observational notes were taken regarding the dynamics of the interview as well as overall thoughts, challenges, and successes. Researchers then met afterwards to debrief on what they were seeing or hearing (e.g., participant’s body language or tone of voice when responding to questions) during the interview. This allowed researchers to more easily adjust their interviewing techniques with the next participant.

Medicolegally, it is important for healthcare teams to be aware of this deficit in future thinking ability as well as perspectives on technology adoption so they can educate their patients and families on cognitive and functional decline early in the disease process; this may include providing an introduction to healthcare technology and appropriate adaptations and modifications to ADL/IADL tasks. In addition, results suggest that people with MCI often cannot imagine the needs of their future self. As such, another important clinical consideration is for the healthcare team to introduce legal documentation that will most likely be required (e.g., healthcare proxy identification, physician orders for life sustaining treatment form [POLST]) at the earliest onset of cognitive decline, prior to the decrease of future thinking abilities. Introducing these early on may provide an opportunity for individuals to have difficult, yet important conversations about their health care goals, values, and preferences with their families, healthcare team, and legal advisors. In turn, these conversations may help individuals in their future thinking ability so that they can better identify and verbalize their wishes.

Continued research adapted for potential deficits in future thinking can inform technological needs for this population as well as interdisciplinary care plans to allow for maximized independence and quality of life for people with dementia and their family members as the disease progresses. By challenging the structure of care, we can meet individuals where they are at and offer a more person-focused intervention to support independence, functional participation, and safety at home. While patient-centered research approaches should continue to include persons with MCI or early ADRD when designing interventions or technology innovations targeting this population, limitations in future thinking ability also need to be taken into account.

## 5. Limitations

Regarding this study’s limitations, we recognize the small sample size may limit the generalizability of the research, and replication of the study with a larger sample size would be beneficial to the current body of research in this area. The observation of impaired future thinking reported and discussed here emerged from the qualitative analysis of the interview results from the parent study; we did not set out to measure episodic future thinking in an experimental context. Therefore, results of formal assessment of specific cognitive domains (e.g., memory, executive function, or attention) and specific measures of episodic future thinking and formal evaluation of insight into the disease process are not available. Similarly, the parent study only included participants with MCI or early ADRD who had caregiver involvement, which may impact participant’s episodic future thinking ability when it comes to imagining the need for assistive technology. Given these limitations, we suggest future studies explore this concept more formally by specifically identifying, separating, and assessing the impact of individual cognitive domains such as attention, memory, and executive function on episodic future thinking as well as the impact of having external support on perceptions of future need.

## Data availability statement

The data analyzed in this study represents a secondary analysis of individually identifiable qualitative interview data that cannot easily be deidentified, and thus is subject to restrictions such that we have no permission to share. Requests to access these datasets cannot be entertained, but other inquiries can be directed to Jaye.McLaren@va.gov.

## Ethics statement

The studies involving human participants were reviewed and approved by VA Bedford Institutional Review Board. The patients/participants provided their written informed consent to participate in this study.

## Author contributions

JM, MS, and LM made substantial contributions to the conception and design of the study and contributed equally to the interpretation of the data and preparation of the manuscript. All authors contributed to the article and approved the submitted version.

## Funding

The work reported in this article was funded by the National Institute on Aging Phase I Small Business Innovation Research #1 R43 AG060781-01 (Principal Investigator: Alvin Ramsey).

## Conflict of interest

The authors declare that the research was conducted in the absence of any commercial or financial relationships that could be construed as a potential conflict of interest.

## Publisher’s note

All claims expressed in this article are solely those of the authors and do not necessarily represent those of their affiliated organizations, or those of the publisher, the editors and the reviewers. Any product that may be evaluated in this article, or claim that may be made by its manufacturer, is not guaranteed or endorsed by the publisher.
